# An evolutionary game study of cockroach control strategies in residential households

**DOI:** 10.1038/s41598-023-33561-w

**Published:** 2023-05-05

**Authors:** Qiuhong Li, Meide Liu, Ting Liu, Ying Tong, Yong Zhang

**Affiliations:** Institute for Disinfection and Vector Control, Beijing Center for Disease Prevention and Control, Beijing, 100013 China

**Keywords:** Entomology, Public health, Preventive medicine

## Abstract

Cockroach control in Beijing's residential households is supported by the local government previously but now it is paid by the residents themselves. Under the new residential household cockroach control strategy, the evolutionary game theory is applied in this study to construct an evolutionary game model for the choice behaviour of both PCO (Pest Control Operation) enterprises and the local governments under government regulation. The evolutionary stabilization strategies under different situations were suggested and the key factors for the evolutionary game behaviour were analyzed through Matlab simulation. It was found that the benefits and costs of the local governments' promotion for the cockroach eradication activities, the incremental benefits of PCO enterprises through government publicity and the subsidies for the activities, and the additional costs of PCO enterprises to participate in cockroach eradication activities are the key factors. The incremental benefits from the publicity of the activities and the government subsidies can be used to incent the PCO enterprises’ activities, which may be failure without the government promotion. This study confirms the decisive role of the strategic choices of PCO enterprises and the government for effective cockroach eradication activities. Therefore, before the campaign is launched, it is necessary to take into account the economic benefits of PCO enterprises and the public interests of the governments so that the game system can evolve out of the "ineffective" and undesirable "locked" state and evolve towards the ideal state, while would be a basis for other anti- pest efforts.

## Introduction

Cockroaches are the most common type of sanitary pest in urban environments^1^. Unlike the blood-sucking arthropods such as mosquitoes and fleas, they are non-blood-feeding insect and can mechanically transmit more than 40 pathogenic microorganisms and parasitic eggs such as bacteria, viruses and moulds^2^. Cockroaches also have both biological transmission mechanisms and their excreta and insects can induce allergic asthma and rhinitis in humans^3^. As negative phototropic insects, they are diurnal and nocturnal. Usually the indoor cockroaches hide in the dark and difficult-to-clean areas such as cracks in tiles, cupboards and cooker hoods, and are often found in places with foods and water. For example, in the kitchens, bathrooms, living rooms and corridors. In China, the German cockroach (*Blattella germanica*), American cockroach (*Periplaneta americana*), black-breasted cockroach (*Periplaneta tuliginosa*), Japanese cockroach (*Periplaneta japonica*), Australian cockroach (*Periplaneta australasiae*) and brown cockroach (*Periplaneta australasiae*) are the six most common species^4^. Cockroaches in Beijing were first found in the food-rich establishments such as hotels and restaurants, and small and medium-sized restaurants. And in recent years, due to the indoor environment improvements, The cockroaches are gradually spreading and affect to other places such as homes, hospitals and schools, and more and more residential households have been infested with cockroaches^5^. It is reported that the cockroaches in households in Beijing are mainly German cockroaches and secondly American cockroaches^6^. Therefore, their controls in residential households becomes an important part of the vector control work in Beijing. Among the integrated cockroach control measurements, the sticky cockroach boards are often used for physical control. Cockroach bait is becoming the most common and indispensable chemical control technique for cockroach control due to its safety, effectiveness and ease of use^7,8^.

It is well known that the cockroaches have strong fertility and it is saying that usually, when residents find a cockroach in one’s homes, there may already be dozens or even hundreds of cockroaches in the house. Thus, once we find cockroaches in our homes, the infestation may be worse. Cockroaches are robust crawlers and can move through sewers and heating duct wells. Based on this habit, it is often challengable to control cockroaches in a single household with promising results^9^. But better cockroache control might be achieved with joint actions from the whole unit, building, and district^10^.

Before the Covid-19 epidemic, the Beijing local government annually organized the city-wide household cockroach extermination campaign involving millions of households and achieved good results. In the early stage of the campaign, each household will be noticed by the community committee and registered if one requires and will cooperate with the visit to one’s homes. Then, the local government will entrust and pay for the cockroach control activities to the Pest Control Operation (PCO) enterprise who will arrangeprofessional technicians to service with sticky cockroach paper or cockroach bait. And during the Covid-19 epidemic, the government funding for the household cockroach control was reduced and it became more challengable to implement widespread household cockroach control activities in line with epidemic protection requirement.

During the Covid-19 epidemic, the travels and tourism are greatly restricted according to the epidemic situation. The extended time residents spend at home may lead to a reduced tolerance for cockroach infestation^11^, thus intensfying the demand of cockroach extermination products and services in residential households. Therefore, during this particular period, the Beijing Municipal Office of Public Health proposed a new strategy to address the demand for widespread cockroach control in the city's residential households. They advocate that residents place their orders for drugs and services for cockroach control, either online or offline, during the cockroach control campaign. Suppose residents purchase their medicines for cockroach control. In that case, they can seek help by calling the government's free technical hotline if they encounter any problems while using the medicines. If it is not convenient for them to do it themself, they can also make an appointment for the PCO enterprises to visit their home. In order to facilitate the purchase of cockroach control products by residents during the unified cockroach control, the government will promote the PCO erterprises to include products suitable for cockroach control in households on the shelves of the unique e-commerce platforms or supermarkets through the promotion of participating companies and the provision of subsidies. If households need professional technicians to visit their homes for cockroach control, the cost will be lower than the market price. This has gradually formed a supply chain of sanitary pest control products and services with the participation of many PCO enterprises^12^. However, to afford the cockroach control drugs and services ty households themselves will inevitably loss their purchasing power and participation and will affect the overall effectiveness of cockroach control in households across the city. Then, during the change in the cockroach control strategy, the governments will promote the change in household consumption and provide subsidies to companies to reduce the purchase cost of households. However, to pay for the cockroach control activities by households is the beginning and how to make use of the government's regulatory measures to attract PCO enterprises' willingness in cockroach control activities with green, environmentally friendly and efficient products as well as high-quality services, become a key issue to implement the new strategy.

Evolutionary games^13,14^ describes a process by which an intelligent body can update its strategy through successive iterations of the game with incomplete and imperfect information, and thus continuously adapts to the external environment and are a powerful tool in the cooperation invesitgation. It contains a macroscopic model with a microscopic foundation, which can more realistically reflect the diversity and complexity of group behaviour. It can be characterized by opposing objectives and strategic interactions, in line with the basic features of the game between PCO enterprises and the government^15^.This promotes the widespread application of this method to analyse the strategy choices of different groups in interactive situations^16^. Currently, the application of evolutionary game theory in solving the problem of division of labour and cooperation involves environmental governance^17–22^, economic trade^23–26^, social and corporate management^27–32^ and many other aspects. Depending on the size structure of the research subjects, theoretical studies are divided into evolutionary games under mixed homogeneous groups and evolutionary games under spatially structured groups to explore the conditions of cooperation dominance, the mechanisms and influencing factors of cooperation under evolutionary stable states, and then make sensible suggestions for practical problems. Some of the most representative teams of scholars include Professor Nowak's team at Harvard University^14,33^ and Professor Traulsen's team^34,35^, and many of their pioneering works are of high theoretical and applied value.

Recently, the evolutionary game theory is used to solve economic and social problems^36^. i.e., the cooperation of relevant actors in supply relationships, mainly between the government and enterprises^37–41^. It shows that this theory can effectively analysize complex and dynamic social problems. With considering the payment changes from local government to households, this study aims to explore the evolutionary mechanism of the game between households and PCO enterprises under the government regulation mechanism so as to promote the implementation of cockroach eradication in households. This study is important for PCO enterprises and the governments to make effective decisions and cockroach eradication strategies.

In this paper, "Basic assumptions and models" section described the theoretical analysis framework and model design of the evolutionary game and discussed the case of the equilibrium point of the game. Meanwhile present the game with simulation results. "Discussion" section described the game simulation alongwith suggestions, and finally, "Conclusions" section summarized this study.

## Basic assumptions and models

### Basic assumptions of evolutionary games

The game is simulated between the local government (after this referred to as 'government') and the PCO enterprises (from now on referred to as 'PCO'). Both of them are finite and rational and adjust and the strategies were adjusted for long-term interaction until an evolutionary equilibrium. The PCO enterprises dominate the supply chain and play an essential role in the effective implementation of local government regulation. The PCO enterprises are for profit maximization while the local governments for maximizing households’ health interests.

Condition 1: In a specific year, a city-wide household cockroach elimination campaign is divided into three stages: pre-publicity planning, organization and implementation, and evaluation. The PCO enterprise is responsible for the implementation and motivate by subsidies from households and local governments.

Condition 2: The PCO enterprise's strategy set is to or not to participate in cockroach control activities (referred to as "to participate" and "not to participate"). The set of strategies from the local governments is to or not to promote cockroach control activities (referred to as "to promote" and "not to promote"), forming a 2 * 2 matrix. The proportion for the participation of the PCO enterprises is set as x and the for no participation is 1 − x. The proportion of government promotion is set as y, and the proportion of non-promotion is 1 − y (0 ≤ y ≤ 1; 0 ≤ x ≤ 1).

Condition 3: Before each large-scaled pest control work is performed, local governments will ask the relevant professional organizations to conduct drug screening for different scenarios and, after careful consideration, choose the best insecticide products for effective control. For household cockroach control activities, the products should be low toxicity, environmental protection and high efficiency. Supposing that the PCO enterprises will "participate in cockroach control activities"., then they should first screen and select low-toxic, environmentally friendly and highly efficient products suitable for sales. If the PCO enterprise provides door-to-door cockroach control services, the price should be slightly lower than the market price and thus, the PCO enterprise's cost is set as Ka. Supposing that the PCO enterprise will not participate in cockroach control activities and the drug screening will not be required. There is no guarantee that the products are suitable for cockroach control in residential households and that they are low-toxic, efficient and environmentally-friendly. The profit gained from selling products and services by the PCO enterprise is set as Wa.

Condition 4: If the PCO enterprise will "participate in cockroach elimination activities", the effectiveness of their products and services will be genuinely evaluated by third parties through questionnaires or positive feedback on the platform, and it will receive a certain amount of local government’s subsidy, S_u_. If the PCO enterprise will not participate cockroach campaign, only profit from selling products will be gained.

Condition 5: If the local governments will 'promotion' the strategy, it will receive benefits, W_b_ (e.g., incentives, subsidies and public recognitions), but will incur promotion costs, C (e.g., providing a free cockroach eradication hotline for residents), and will also have to pay subsidies for PCO enterprises. If the local governments will not promote the strategy, no costs or benefits will be mentioned. However, if a city-wide household cockroach eradication campaign is not initiated, i.e., both the PCO enterprise and local government will not implement the campaign, the local government might be complainted or requested by the public for dealing with cockroach infestation and the corresponding costs is set as F_c_.

### Models and solution

Based on the above assumptions the evolutionary game payment matrix is shown in Table 1.

**Table 1:** New resident household cockroach eradication strategy under the local government-PCO payment matrix.

	Government	
	Promote y	Don’t promote 1-y
PCO		
Participate in cockroach control activities x	(W_a_ − K_a_ + Q_a_ + S_u_,W_b_ − C − S_u_)	(W_a_ − K_a_ + Q_a_,0)
Do not participate in cockroach control activities 1 − x	(W_a_ − K_b_,W_b_ − C)	(W_a_ − K_b_, − F_c_)

#### Equilibrium point solving for evolutionary games

From Table 1, the expected benefits from local government promotion activities are

1 $${\text{E1}} = {\text{x}}({\text{W}}_{{\text{b}}} - {\text{C}} - {\text{S}}_{{\text{u}}} ) + \left( {{1} - {\text{x}}} \right)({\text{W}}_{{\text{b}}} - {\text{C}}) = {\text{W}}_{{\text{b}}} - {\text{C}} - {\text{xS}}_{{\text{u}}}$$

The expected benefits without local government’s promotion activities are

2 $${\text{E2}} = \left( {{1} - {\text{x}}} \right)( - {\text{F}}_{{\text{c}}} ) = {\text{xF}}_{{\text{c}}} - {\text{F}}_{{\text{c}}}$$

Then, the average return to the local government is

3 $${\overline{\text{E}}} = {\text{yE}}1 + \left( {1 - {\text{y}}} \right){\text{E}}2$$

Similarly, the expected benefit of the PCO enterprise's participation in the cockroach eradication campaign is

4 $${\text{U1}} = {\text{y}}\left( {{\text{W}}_{{\text{a}}} - {\text{K}}_{{\text{a}}} + {\text{Q}}_{{\text{a}}} + {\text{S}}_{{\text{u}}} } \right) + \left( {{1} - {\text{y}}} \right)\left( {{\text{W}}_{{\text{a}}} - {\text{K}}_{{\text{a}}} + {\text{Q}}_{{\text{a}}} } \right) = {\text{W}}_{{\text{a}}} - {\text{K}}_{{\text{a}}} + {\text{Q}}_{{\text{a}}} + {\text{yS}}_{{\text{u}}}$$

The expected benefits without the PCO enterprise’s participation are

5 $${\text{U2}} = {\text{y}}\left( {{\text{W}}_{{\text{a}}} - {\text{K}}_{{\text{b}}} } \right) + \left( {{1} - {\text{y}}} \right)\left( {{\text{W}}_{{\text{a}}} - {\text{K}}_{{\text{b}}} } \right) = {\text{W}}_{{\text{a}}} - {\text{K}}_{{\text{b}}}$$

The average earnings of the PCO enterprises are

6 $${\overline{\text{U}}} = {\text{xU}}1 + \left( {1 - {\text{x}}} \right){\text{U}}2$$

The replication dynamics equations for the PCO enterprises and local governments, respectively, are

7 $${\text{F}}\left( {\text{x}} \right) = {\text{dx}}/{\text{dt}} = {\text{x}}({\text{U1 }} - {\overline{\text{U}}}) = {\text{x}}\left( {{1} - {\text{x}}} \right)\left( {{\text{U1}} - {\text{U2}}} \right) = {\text{x}}\left( {{1} - {\text{x}}} \right)\left( {{\text{K}}_{{\text{b}}} - {\text{K}}_{{\text{a}}} + {\text{Q}}_{{\text{a}}} + {\text{yS}}_{{\text{u}}} } \right)$$

8 $${\text{F}}\left( {\text{y}} \right) = {\text{dy}}/{\text{dt}} = {\text{y}}({\text{E1 }} - {\overline{\text{E}}}) = {\text{y}}\left( {{1} - {\text{y}}} \right)\left( {{\text{E1}} - {\text{E2}}} \right) = {\text{y}}\left( {{1} - {\text{y}}} \right)\left( {{\text{W}}_{{\text{b}}} + {\text{F}}_{{\text{c}}} - {\text{C}} - {\text{xS}}_{{\text{u}}} - {\text{xF}}_{{\text{c}}} } \right)$$

Let dx/dt = 0 and dy/dt = 0, the five equilibrium points for the interaction of the two strategies can be derived as (0,0),(0,1),(1,0),(1,1),(M, N). (0 ≤ M ≤ 1,0 ≤ N ≤ 1). Then,

9 $${\text{M}} = \left( {{\text{W}}_{{\text{b}}} + {\text{F}}_{{\text{c}}} - {\text{C}}} \right)/\left( {{\text{F}}_{{\text{c}}} + {\text{S}}_{{\text{u}}} } \right)$$

10 $${\text{N}} = \, \left( {{\text{K}}_{{\text{a}}} - {\text{K}}_{{\text{b}}} - {\text{Q}}_{{\text{a}}} } \right)/{\text{S}}_{{\text{u}}}$$

#### Stability analysis

Since the equilibrium point derived by replicating the dynamic equations is not necessarily the system's evolutionary stability strategy (ESS), the system is analyzed for local stability and the stability of the evolutionary equilibrium point is derived using the Jacobian matrix (denoted as J).

11 $$J = \left[ {\begin{array}{*{20}c} {\partial F\left( x \right)/\partial x} & {\partial F\left( x \right)/\partial y} \\ {\partial F\left( y \right)/\partial x} & {\partial F\left( y \right)/\partial y} \\ \end{array} } \right] = \left[ {\begin{array}{*{20}c} {a_{11} } & {a_{12} } \\ {a_{21} } & {a_{22} } \\ \end{array} } \right]$$

where a_11_, a_12_, a_21_ and a_22_ are respectively

12 $${\text{a}}_{{{11}}} = \left( {{1} - {\text{2x}}} \right)({\text{K}}_{{\text{b}}} - {\text{K}}_{{\text{a}}} + {\text{Q}}_{{\text{a}}} + {\text{yS}}_{{\text{u}}} )$$

13 $${\text{a}}_{{{12}}} = {\text{x}}\left( {{1} - {\text{x}}} \right){\text{S}}_{{\text{u}}}$$

14 $${\text{a}}_{{{21}}} = {\text{y}}\left( {{1} - {\text{y}}} \right)( - {\text{S}}_{{\text{u}}} - {\text{F}}_{{\text{c}}} )$$

15 $${\text{a}}_{{{22}}} = \left( {{1} - {\text{2y}}} \right)\left( {{\text{W}}_{{\text{b}}} + {\text{F}}_{{\text{c}}} - {\text{C}} - {\text{xS}}_{{\text{u}}} - {\text{xF}}_{{\text{c}}} } \right)$$

If both conditions are satisfied, the equilibrium point for replicating the dynamic equations is the Evolutionary Stability Strategy (ESS).

1. 16 $$trJ = a_{11} + a_{22} < 0$$
2. 17 $$\det J = \left[ {\begin{array}{*{20}c} {a_{11} } & {a_{12} } \\ {a_{21} } & {a_{22} } \\ \end{array} } \right] = a_{11} a_{22} - a_{12} a_{21} > 0$$

Therefore, the specific values of a_11_, a_12,_ a_21_ and a_22_ at the five local equilibrium points are derived, as shown in Table 2.

18 $${\text{P}} = {\text{S}}_{{\text{u}}} ({\text{W}}_{{\text{b}}} + {\text{F}}_{{\text{c}}} - {\text{C}})\left( {{\text{S}}_{{\text{u}}} - {\text{W}}_{{\text{b}}} + {\text{C}}} \right)/\left( {{\text{F}}_{{\text{c}}} + {\text{S}}_{{\text{u}}} } \right)^{{2}}$$

19 $${\text{Q}} = \left( {{\text{S}}_{{\text{u}}} + {\text{Q}}_{{\text{a}}} + {\text{K}}_{{\text{b}}} - {\text{K}}_{{\text{a}}} } \right)\left( { - {\text{K}}_{{\text{a}}} + {\text{K}}_{{\text{b}}} + {\text{Q}}_{{\text{a}}} } \right)({\text{S}}_{{\text{u}}} + {\text{F}}_{{\text{c}}} )/{\text{S}}_{{\text{u}}}^{{2}}$$

**Table 2:** The value of a_11_, a_12_, a_21_, and a_22_ at the equilibrium point.

Equilibrium point	a_11_	a_12_	a_21_	a_22_
(0,0)	K_b_ − K_a_ + Q_a_	0	0	W_b_ + F_c_ − C
(0,1)	K_b_ − K_a_ + Q_a_ + S_u_	0	0	− (W_b_ + F_c_ − C)
(1,0)	−(K_b_ − K_a_ + Q_a_)	0	0	W_b_ − C − S_u_
(1,1)	− (K_b_ − K_a_ + Q_a_ + S_u_)	0	0	− (W_b_ − C − S_u_)
(M, N)	0	P	Q	0

where the specific expressions for P and Q are, respectively.

At the point (M, N), if a_11_ + a_22_ = 0, it will not satisfy the condition: trJ < 0. Thus, it is only necessary to analyze the stability of the remaining four equilibrium points.

Case I: ESS is (0,0) when Q_a_ < K_a_ − K_b_ < S_u_ + Q_a_ and W_b_ + F_c_ < C, or S_u_ + Q_a_ < K_a_ − K_b_ and W_b_ + F_c_ < C (see Table 3).

**Table 3:** The stability of the equilibrium point of case 1.

Equilibrium point	trJ	detJ	Local stability
(0,0)	−	+	ESS
(0,1)	Uncertain	−	Saddle point
(1,0)	Uncertain	+	Unstable point
(1,1)	+	−	Saddle point

Case 2: When Q_a_ < K_a_-K_b_ < S_u_ + Q_a_ and C − F_c_ < W_b_ < C + S_u_, there is no evolutionary stabilization strategy for the system (see Table 4).

**Table 4:** The stability of the equilibrium point of case 2.

Equilibrium point	trJ	detJ	Local stability
(0,0)	Uncertain	−	Saddle point
(0,1)	Uncertain	−	Saddle point
(1,0)	Uncertain	−	Saddle point
(1,1)	Uncertain	−	Saddle point

Case 3: When S_u_ + Q_a_ < K_a_ − K_b_ and W_b_ > C + S_u_, or S_u_ + Q_a_ < K_a_ − K_b_ and C − F_c_ < W_b_ < C + S_u_, the ESS is (0,1) (see Table 5).

**Table 5:** The stability of the equilibrium point of case 3.

Equilibrium point	trJ	detJ	Local stability
(0,0)	Uncertain	−	Saddle point
(0,1)	−	+	ESS
(1,0)	Uncertain	+	Saddle point
(1,1)	+	−	Saddle point

Case 4: ESS is (1,0) when Q_a_ > K_a_ − K_b_ and C − F_c_ < W_b_ < C + S_u_, or Q_a_ > K_a_ − K_b_ and W_b_ < C − F_c_;

Case 5: The ESS is (1,1) when Q_a_ < K_a_ − K_b_ < S_u_ + Q_a_ and W_b_ > C + S_u_, or Q_a_ > K_a_ − K_b_ and W_b_ > C + S_u_. The same can be proved for the equilibrium point stability method for cases 4 and 5.

### Analysis of evolutionary simulation results

Matlab was used to assign values to critical data. In order to make more scientific and objective simulation results. The parameters below are assigned randomly under above assumptions in the previous section. They do not represent the value of payments or revenues from the local government and the PCO enterprise in the realistic Beijing household cockroach eradication activities. The values can be set with actual situation for different pest control activities.

#### Validation of evolutionary stability strategy scenario

##### Situation (1)

**Figure 1:**
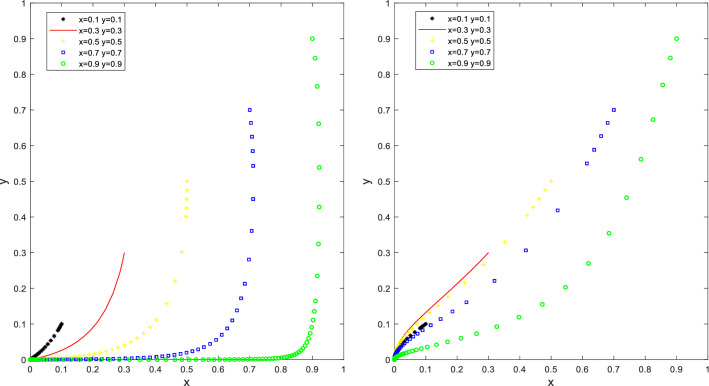
Simulation results of the evolution of the stable point (0,0) of situation 1.

Let Q_a_ = 30, S_u_ = 20, K_a_ = 60, K_b_ = 20, F_c_ = 10, W_b_ = 30, C = 50 to satisfy the first condition Q_a_ < K_a_ − K_b_ < S_u_ + Q_a_ and W_b_ + F_c_ < C, and let Q_a_ = 30, S_u_ = 10, K_a_ = 70, K_b_ = 20, F_c_ = 10, W_b_ = 30, C = 50 to satisfy the second condition S_u_ + Q_a_ < K_a_ − K_b_ and W_b_ + F_c_ < C, the simulation results are shown in Fig. 1. With different initial ratios of x and y, as the number of steps of evolutionary iteration increases, the ratio of the local governments’ promotion and the ratio of the PCO enterprises participation are decreased, finally forming a stable point (0,0). (The PCO enterprises do not participate in cockroach eradication activities and the local government does not promote the cockroach eradication activities).

As shown in Fig. 1, many governments initially chose the "promotion" strategy to maximize social benefits and mobilize PCO enterprises to participate in cockroach eradication activities. However, since the cost of planning and implementing the activities is higher than the benefit, and dealing with complaints from households is lower, the potential benefit of promoting the campaign is lower than the potential benefit if the campaign is not promoted. The local governments will eventually be driven by their interests to "promote" the activities and tend to "not promote" it. A significant proportion of PCO enterprises initially opted for the "participate in cockroach eradication" strategy. However, the incremental advertising benefits were less than the reduced costs of the "do not participate in cockroach eradication" strategy. The local government's response to the "participate in cockroach eradication" strategy was not as effective. However, since the incremental revenue from advertising is less than the cost reduction from choosing the "no cockroach campaign" strategy. The local government provides fewer subsidies to PCO enterprises participating in the cockroach campaign. The net benefit of choosing the "participate in cockroach campaign" strategy is less than the net benefit of choosing the "not participate in cockroach campaign" strategy. Eventually, PCO enterprises are driven by their interests to choose the "participate in cockroach campaign" strategy. In the end, PCO enterprises are driven by their interests, from "participate in cockroach campaign" to "not to participate in cockroach campaign". Under such circumstances, the local governments will not pay for cockroach control activities. PCO enterprises are unwilling to participate in cockroach control activities, so the price of cockroach control products in the market is the highest, the effectiveness is the worst, and the overall benefits to both parties are minimal, which ultimately leads to the failure of cockroach control activities.

##### Situation (2)

**Figure 2:**
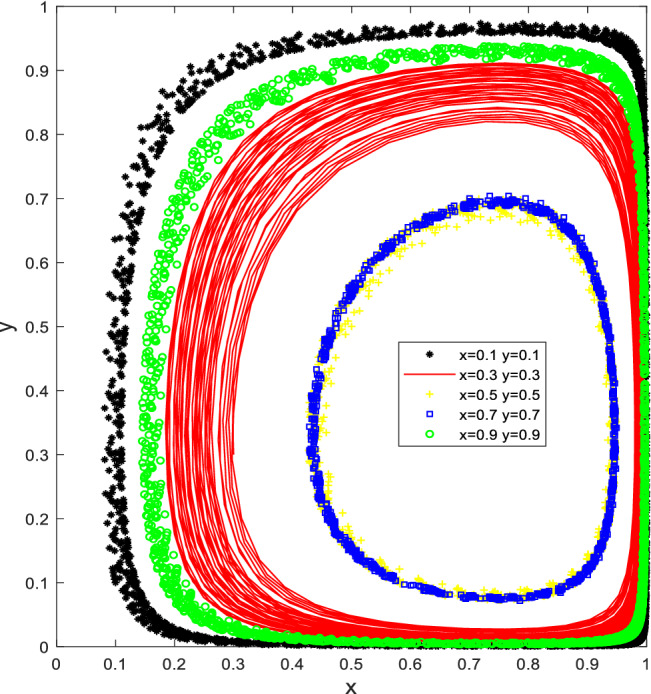
Simulation results for scenario 2 without stable point evolution.

Let Q_a_ = 30, S_u_ = 30, K_a_ = 70, K_b_ = 30, F_c_ = 10, W_b_ = 50, C = 30 to satisfy the condition Q_a_ < K_a_ − K_b_ < S_u_ + Q_a_ and C − F_c_ < W_b_ < C + S_u_, the simulation results of its evolutionary model are shown in Fig. 2. The proportion of governments promoting cockroach eradication activities in households and the proportion of PCO enterprises participating in cockroach eradication activities have been changing. However, the trend of the evolutionary strategy of the two behaviours is generally consistent, and there is no evolutionary stability strategy (ESS) in the decision-making behaviour between the local governments and PCO enterprises.

As shown in Fig. 2, for different initial ratios of x and y, according to the number of evolutionary iterations, when the cost savings of the PCO enterprise choosing the "not to participate in cockroach campaign" strategy is greater than the incremental revenue caused by the advertising effect obtained by choosing the "participate in cockroach campaign" strategy, and the incremental revenue is less than the sum of the subsidy obtained by the PCO enterprise and the "participate in cockroach campaign" strategy, the PCO enterprise chooses the "participate in cockroach campaign" strategy when the local government promotes the activities and the "not to participate in cockroach activities" strategy when it does not promote the activities. Suppose the incremental benefit is less than the sum of the incremental benefit and the subsidy received from participating in the cockroach campaign. In that case, the PCO enterprise chooses the "Participate in cockroach campaign" strategy when the local government promotes the activities and the "not participating in cockroach campaign" strategy when it does not promote it. When the benefits of the local governments from cockroach control activities in households are more significant than the difference between the cost of promotion and the cost of handling complaints and requests for assistance and less than the sum of the cost of promotion and the subsidies provided to PCO enterprises, the behaviour of PCO enterprises and the local governments evolves similarly, with no stable evolutionary strategy. This is consistent with the reality that the local governments actively promote the initiation of city-wide cockroach campaigns when the cockroach infestation is severe and do not want to initiate cockroach campaigns when the infestation is not severe.

##### Situation (3)

**Figure 3:**
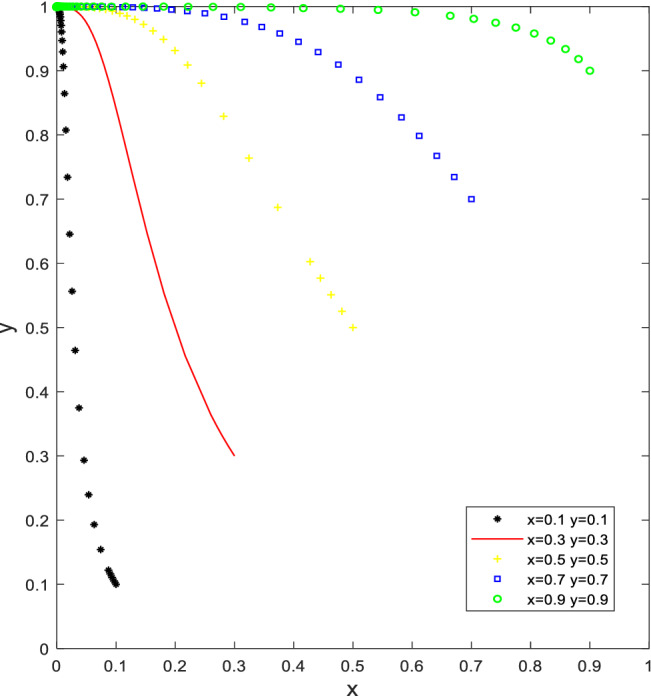
Simulation results of the evolution of the stable point (0,1) of scenario 3.

Let Q_a_ = 20, S_u_ = 10, K_a_ = 70, K_b_ = 30,W_b_ = 50, C = 30 to satisfy the first condition S_u_ + Q_a_ < K_a_ − K_b_ and W_b_ > C + S_u_, or let Q_a_ = 20, S_u_ = 20, K_a_ = 70, K_b_ = 20, F_c_ = 20, W_b_ = 40, C = 30 to satisfy the second condition S_u_ + Q_a_ < K_a_ − K_b_ and C − F_c_ < W_b_ < C + S_u_ (as in Fig. 3). As the number of evolutionary iterations increases, the proportion of the local governments promoting activities increases and the proportion of PCO enterprises participating in cockroach eradication activities decreases, forming an evolutionary equilibrium point (0,1). (PCO enterprises do not participate in cockroach eradication activities and the governments promote cockroach eradication activities).

In this case, it can be seen that a higher proportion of PCO enterprises initially opted for the "participate in cockroach campaign" strategy, but since the sum of the total subsidy and advertising received was less than the cost reduction of the "not participating in cockroach campaign" strategy, the net benefit of choosing the "participate in cockroach campaign" strategy was less than the net benefit of choosing the "not participating in cockroach control" strategy. The net benefit of the "participate in cockroach campaign" strategy is less than that of the " not participating in cockroach campaign" strategy. Ultimately, PCO enterprises were driven by their interests to maximize their profits and tended to switch from the "participate in cockroach campaign" to the "not participating in cockroach campaign" strategy. At the same time, a higher proportion of governments initially chose the "do not promote" strategy. "As the proportion of PCO enterprises choosing "not participating in cockroach campaign" increases, the cost of promotion by the governments decreases, making the benefits of promotion by the governments smaller than the benefits of not promoting. Therefore, governments tend to move from "not promoting" to "promoting" to pursue their interests. Even though the governments actively promote, they can only promote PCO enterprises' participation in cockroach extermination activities in the short term. However, they cannot fundamentally promote PCO enterprises' willingness to participate in cockroach extermination activities to provide quality and efficient products and services and guarantee the effectiveness of cockroach extermination in residential households.

##### Situation (4)

**Figure 4:**
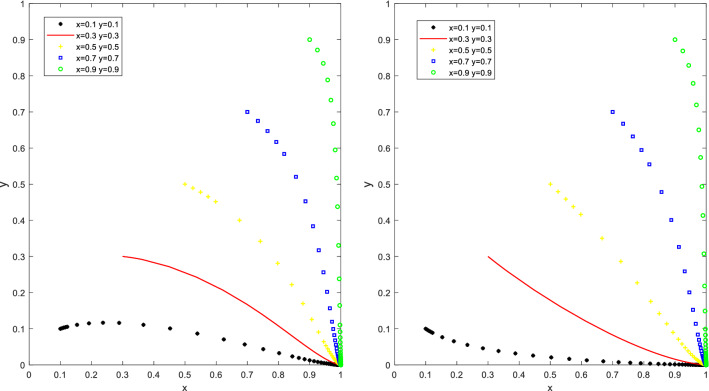
Simulation results of the evolution of the stable point (1,0) of situation 4.

Let Q_a_ = 50, S_u_ = 10, K_a_ = 50, K_b_ = 20, F_c_ = 30, W_b_ = 30, C = 50 to satisfy the first condition Q_a_ > K_a_ − K_b_ and C − F_c_ < W_b_ < C + S_u_, or let Q_a_ = 50, K_a_ = 50, K_b_ = 20, F_c_ = 10, W_b_ = 30, C = 50 to satisfy the second condition Q_a_ > K_a_ − K_b_ and W_b_ < C − F_c_ (as in Fig. 4). With different initial proportions of x and y, many PCO enterprises chose the "not participating in cockroach campaign" strategy as the number of evolutionary iterations increased. However, as the incremental benefits from the advertising benefits of choosing the "participate in cockroach campaign" strategy outweighed the cost reduction of choosing the "not participating in cockroach campaign" strategy, the benefits of choosing the "participating in cockroach campaign" strategy outweighed the benefits of choosing the "not participating in cockroach campaign" strategy. In the end, PCO enterprises are driven by the desire to maximize their profits by switching from "not participating in cockroach campaign" to "participate in cockroach campaign". However, as the cost of promotion is higher than the benefit, the local governments are eventually driven by their interests from "promote" to "not promote". If the local governments do not promote for a long period, the funding for cockroach eradication activities for city households may be ineffective. The social benefits PCO enterprises gain from participating in cockroach eradication activities will outweigh the increased costs of participating in cockroach eradication activities. PCO enterprises will be willing to participate in cockroach eradication activities even if the local government does not provide subsidies.

##### Situation (5)

**Figure 5:**
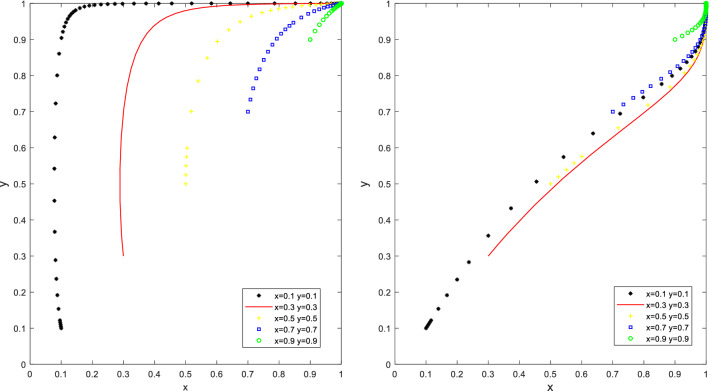
Simulation results of the evolution of the stable point (1,1) of situation 5.

Let Q_a_ = 40, S_u_ = 20, K_a_ = 70, K_b_ = 20, W_b_ = 60, C = 30 to satisfy the first condition Q_a_ < K_a_ − K_b_ < S_u_ + Q_a_ and W_b_ > C + S_u_, or let Q_a_ = 50, S_u_ = 10, K_a_ = 50, K_b_ = 20, F_c_ = 10, W_b_ = 50, C = 30 to satisfy the second condition Q_a_ > K_a_ − K_b_ and W_b_ > C + S_u_. The stable point for the behavioural evolution of PCO enterprises and the local governments is (1,1). (PCO enterprises participate in cockroach eradication activities and the local governments promote the activities). The results of the simulation are shown in Fig. 5, at different initial proportions of x and y, the proportion of PCO enterprises participating in cockroach extermination activities and the proportion of the local governments promoting activities increase according to the number of steps of evolutionary iterations.

PCO enterprises initially have a higher percentage of subsidies to choose the "not participating in cockroach campaign" strategy. "The incremental benefits are greater than the sum of the incremental benefits and the subsidies received, or the incremental benefits are greater than the cost reduction of the "participate in cockroach campaign" and "not participating in cockroach campaign" strategies. The benefits of the "participate in cockroach campaign" strategy are greater than the benefits of the "not participate in cockroach campaign" strategy. Ultimately, the PCO enterprises shift from a "not participate in cockroach campaign" to a "participate in cockroach campaign". As the cost of promotion decreases, the benefits of "promotion" are less than the benefits of "non-promotion". As a result, the local governments, driven by the desire to maximize their interests, shift from 'not promote' to 'promote'. This suggests that government incentives through promotion and subsidies to PCO enterprises are in effect. As the cost of promotion decreases and the benefits of promotion increase, the governments actively promote cockroach eradication activities until the interaction evolves to an equilibrium point (1,1).

### Effect of changes in model parameters on the direction of evolution

This paper aims to facilitate the evolution of both sides to the optimal model of cockroach eradication activities in the city's households. to facilitate the eventual evolution of the game between the two sides to the ideal state where PCO enterprises choose to participate in cockroach eradication activities and the local governments promote them (x = 1, y = 1).

**Figure 6:**
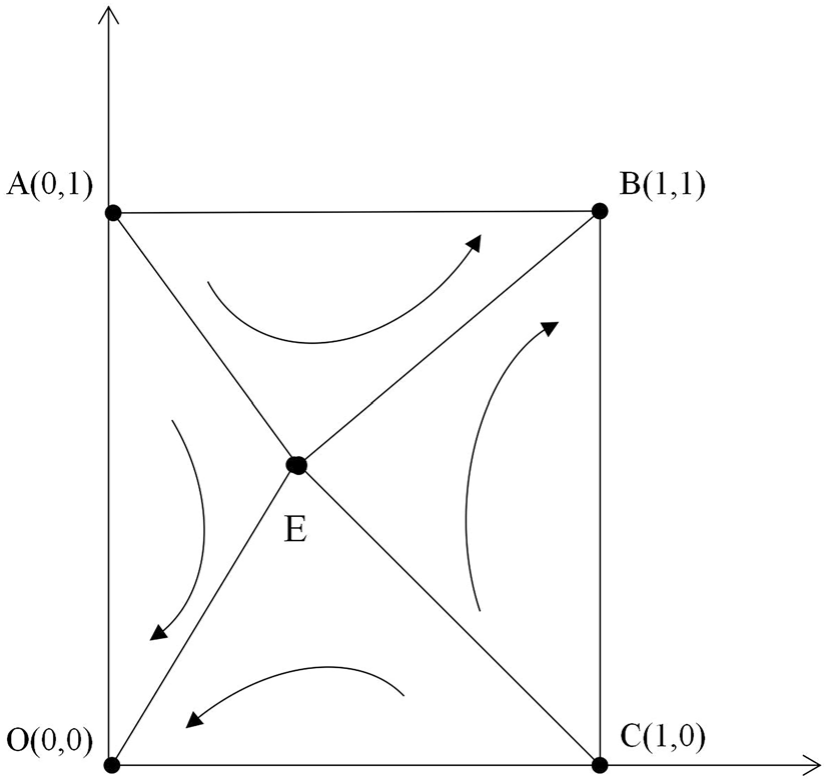
Evolutionary phase diagram of the two sides of the game.

As the diagram shows, the system has been in a gradual evolutionary process, and the final evolutionary path depends on the initial state of the system and the game payout matrix. Therefore, the position of point E determines the probability of the system evolving from entirely negative to wholly positive. As in Fig. 6.When the area of quadrilateral ABCE is greater than the area of quadrilateral AECO, the probability of the system converging to (1,1) is greater than the probability of converging to (0,0)(see Table 6). The formula for the area of quadrilateral ABCE.

$$1 - \frac{1}{2}\left( {\frac{{W_{b} - F_{c} - C}}{{F_{c} + S_{u} }} + \frac{{k_{a} - K_{b} - Q_{a} }}{{S_{u} }}} \right)$$

**Table 6:** The influence of parameter changes on the evolution direction between PCO enterprise and government.

Parameter change	The change in area of quadrilateral ABCD	Direction of evolution
W_b_↓	↑	Participate in cockroach control activities, promote cockroach control activities
F_c_↓	↑	Participate in cockroach control activities, promote cockroach control activities
C↑	↑	Participate in cockroach control activities, promote cockroach control activities
S_u_↑	↑	Participate in cockroach control activities, promote cockroach control activities
K_a_↓	↑	Participate in cockroach control activities, promote cockroach control activities
K_b_↑	↑	Participate in cockroach control activities, promote cockroach control activities
Q_a_↑	↑	Participate in cockroach control activities, promote cockroach control activities

## Discussion

In this paper, we construct an evolutionary game model of whether PCO enterprises are willing to participate in cockroach control activities, whether the local governments promote the activities, and establish dynamic replication equations and evolutionary stabilization strategies for PCO enterprises and the local governments. The study concluded that: the benefits and costs of the local government promotion of cockroach eradication activities, the incremental benefits of government promotion of cockroach eradication activities to PCO enterprises and the subsidies for the activities, and the increased costs for PCO enterprises to participate in cockroach eradication activities are the key factors influencing the evolutionary game behaviour of PCO enterprises and the local governments; the increase of the local government subsidies for PCO enterprises help to promote their participation in cockroach eradication activities; the increase of benefits of the local government promotion of cockroach eradication activities and The increase in government subsidies for PCO enterprises can help promote their participation in cockroach control activities. However, without the local governments’ promotion, using subsidies to incentivize PCO enterprises to participate in activities would be ineffective. Thus, in the early stages of the transition from government-paid to individually paid household cockroach control activities in the city, there is a great deal of uncertainty about the benefits to PCO enterprises compared to before, which inevitably leads to low participation by PCO enterprises. The model's findings suggest that PCO enterprises will be willing to participate in cockroach control activities when their incremental benefits are large enough, even if they are not subsidized. It is then necessary for the local governments to take active promotion measures (increasing the popularization of cockroach elimination activities, helping enterprises to pass on incremental benefits.) to increase the motivation of PCO enterprises to participate in cockroach elimination activities. In summary, the following recommendations are made in this paper:

Under the new household cockroach control strategy, the relevant governments should strengthen the publicity of household cockroach control activities and actively cultivate residents' consumption awareness. Firstly, through diversified publicity channels and strategies to raise residents' level of awareness of cockroach extermination activities, promote the expected results of residents' household cockroach extermination activities, facilitate the transformation of residents' awareness from price consumption to value consumption, and promote the realization of the concept of consumption for cockroach extermination. Secondly, combined with the government's effective regulation mechanism, it will promote incremental revenue through publicity to achieve synergy and unity between residents' households and PCO enterprises' participation in cockroach extermination activities and ultimately achieve good results in cockroach control in residents' households. Once again, the local government can help PCO enterprises to reduce costs through measures such as tax reduction.

At the initial stage of cooperation between PCO enterprises and the local government, the initial psychological expectations of both sides have an important impact on the choice of subsequent cooperation strategy. PCO enterprises should also strengthen the atmosphere of active participation in household cockroach eradication by widely publicizing and minimizing the purchase costs for residents. Firstly, PCO enterprises should promote the concept of household cockroach extermination through advertising, packaging and other forms of marketing to popularise knowledge of cockroach extermination. Secondly, we should improve the information technology of product management in the supply chain and strengthen the display of products. As the public's awareness of environmental protection increases, residential households gradually pursue less toxic, environmentally friendly and efficient products. By establishing an information system for cockroach control products, consumers can check information on the active ingredients and killing speed of products in real-time, which is conducive to enhancing consumers' product purchase preferences. At the same time, PCO enterprises should also follow this direction to improve the quality of cockroach control products in the market and form a good supply and sales cycle. Thirdly, PCO enterprises should strengthen the R&D and innovation of product technology, integrate and optimize resources, minimize product costs and improve product and service quality. They should also set reasonable prices for the products they sell and create an excellent consumer environment to promote consumption by residents and maximize the utility of consumers and the profitability of the supply chain.

In the current economic environment, online shopping has become mainstream for residents. Third-party evaluation agencies should therefore consider adding reviews from users of e-commerce platforms when evaluating PCO enterprises for acceptance and collecting timely feedback from users on the effectiveness of their products. It will help the regulatory authorities monitor the quality of medicines and services sold by PCO enterprises, and PCO enterprises with more sales and positive reviews on their platforms should be given more subsidies to increase the motivation of PCO enterprises to improve the quality of their products and services.

Based on the above findings, the role of comprehensive science and technology promotion is most important, which will drive the participation of residential households and PCO enterprises in the city's cockroach eradication activities. The supply chain of preferential products and services that the local government has secured through measures such as subsidies and tax breaks will also facilitate the transformation of the strategy of cockroach eradication in residential households.

## Conclusions

In this paper we construct an evolutionary game model to analyse the behavioural decisions of PCO enterprises and the government in a residential household cockroach eradication campaign under different benefits, costs and subsidy measures. First, five possible evolutionary scenarios and five possible evolutionary outcomes are given, and then the conditions necessary to obtain the different evolutionary outcomes are summarised. Numerical simulations are also carried out in order to verify our conclusions and to make our model analysis more understandable. Finally, the influence of different factors on the direction of evolution of both sides is analysed. The construction of the model is useful in helping the governments to formulate relevant policies based on the expected outcomes, and it can also motivate PCO enterprises to participate in cockroach eradication activities under different scenarios. In addition, it can also help PCO enterprises and the governments to establish a healthy long-term partnership for vector control and safeguard urban public health construction. This experience can also be replicated in the work of urban residents who are in need of rodent and mosquito and fly control.

Limitations of this study include the comprehensiveness of the game model of household cockroach eradication strategies and the lack of precision in the analysis of actual random factors. Some numerical experimental data from empirical values. Future work will address these issues by constructing a three-party game including households and analysing the stochastic factors to make the results more objective and usable.

## Data Availability

All data generated or analysed in this study are included in this published article. Datasets used and analysed in the current study are available upon reasonable request from the corresponding author.
